# Innovation through recycling in Iron Age plaster technology at Tell el-Burak, Lebanon

**DOI:** 10.1038/s41598-025-05844-x

**Published:** 2025-07-07

**Authors:** Silvia Amicone, Adriano Orsingher, Emma Cantisani, Sara Calandra, Kamal Badreshany, Cynthianne Spiteri, Christoph Berthold, Hélène Sader, Aaron Schmitt, Jens Kamlah

**Affiliations:** 1 Archaeometry Research Group (CCA-BW), Eberhard Karls University Tübingen, Wilhelmstr. 56, 72074 Tübingen, Germany; 2 Institute of Archaeology, University College London, 31–34 Gordon Square, London, WC1H 0PY UK; 3 Institute of Biblical Archaeology, Eberhard Karls University Tübingen, Liebermeisterstr. 14, 72076 Tübingen, Germany; 4 Department of Prehistory, Ancient History and Archaeology, Complutense University of Madrid, C/Prof. Aranguren, s/n, 28040 Madrid, Spain; 5 Institute of Heritage Science, CNR, via Madonna del Piano 10, 50019 Sesto Fiorentino, Italy; 6 Department of Earth Sciences, University of Florence, via la Pira 4, 50121 Florence, Italy; 7 Department of Archaeology, Durham University, South Road, Durham, DH1 3LE UK; 8 ArchaeoBiomics Laboratory, Department of Life Sciences and Systems Biology, University of Turin, Via Accademia Albertina 13, 10123 Torino, Italy; 9 Department of History and Archaeology, American University of Beirut, Riad el Solh, P.O. Box 11-0236, Beirut, 1107 2020 Lebanon; 10 Institut für Ur- und Frühgeschichte und Vorderasiatische Archäologie, Heidelberg University, Sandgasse 7, 69117 Heidelberg, Germany

**Keywords:** Plaster, Iron Age technology, Ceramics, Recycling, Lebanon, Phoenician, Ecology, Materials science

## Abstract

Recent excavations at the Phoenician coastal site of Tell el-Burak, a large-scale agricultural production centre in use during 725–350 BCE, have uncovered the first Iron Age wine press in Lebanon. This discovery enabled a systematic, interdisciplinary study of its plaster, offering insights into ancient construction technologies. The analysis extended to two other plastered installations found in separate rooms within the complex, allowing for a comprehensive comparison. An integrated program of archaeometric analysis, including optical microscopy in polarising light, X-ray powder diffraction, scanning electron microscopy, thermogravimetry, and organic residue analysis, was applied to samples from these three structures to investigate plaster composition and technological variability. Previous studies identified the use of crushed ceramic fragments in a lime-based plaster. Now, new analyses provide deeper insights into the nature of the binder, revealing how the addition of ceramic sherds may have enhanced the plaster’s mechanical properties and hydraulicity, making it a significant early example of hydraulic mortar. These findings confirm the existence of a local, innovative tradition of lime-plaster manufacture in southern Phoenicia. This investigation not only sheds light on the specific technological practices of Tell el-Burak, but also contributes to the broader understanding of Phoenician and Punic technological advancements in the Iron Age Mediterranean.

**Supplementary Information:**

The online version contains supplementary material available at 10.1038/s41598-025-05844-x.

## Introduction

The appearance of hydraulic mortars is a crucial step in the development of lime-based mortar technology^1^. Hydraulic mortars are composed of binders capable of setting even in the presence of water or high humidity. They offer superior mechanical performance and durability compared to air-hardening mortars. For this reason, they are particularly suitable for structures exposed to moisture or in direct contact with water, such as aqueducts, bridges, canals, dykes, and cisterns. This type of material, in ancient times, is achieved by burning limestone containing 5 to 20% clay or siliceous limestone or by mixing air-hardening lime with Si, Al rich aggregates that are partially or totally reactive and generally defined as “pozzolanic material”^2^. The latter normally reacts with the highly alkaline environment created in the lime-saturated water and induce the dissolution of the silicate or aluminosilicate phases and the subsequent precipitation of insoluble Si-rich hydrate phases^3^. The reactive pozzolanic material may be natural or synthetic, and may include silica glass, volcanic ash, radiolarite, diatomaceous earth, phytoliths, ceramics, clay, metallurgical slags, or other reactive aluminosilicate compound. The inclusion of pozzolanic material significantly improves the chemical bonding between lime and aggregates, yielding a dense, cohesive binder with increased water resistance and enhanced structural performance^2,4,5^.

Mortars with pozzolanic material were particularly utilised in Roman architecture^6–11^. An early example which provided evidence of lime mortar mixed with volcanic ash dates back to the Pre-Pottery Neolithic B (PPNB) and is found at the site of Aşıklı Höyük in Turkey, but this probably happened accidentally according to the authors^12^. More clear examples of this type of mortar can be found in the Greek and Aegean world in the 2nd millennium BCE (^1^ and literature therein).

An unclear trajectory of adoption and diffusion of hydraulic mortar technology occurs between the Aegean tradition and the standardised use of this type of mortar by Romans, through ideas originating from the Eastern Mediterranean spreading westward during the early Iron Age likely via the Phoenicians. The latter are reported to have used plasters and mortars^3^ obtained by mixing crushed tiles or ceramic with lime (the so-called cocciopesto), a tradition attested only rarely across the Mediterranean before the Roman period^12–14^.

The preliminary analysis of the plastered installation connected to wine production at the site of Tell el-Burak showed what could be the earliest concrete evidence of this technique in the Phoenician and Punic Mediterranean world^15^. The archaeometric investigation that combined both micro X-ray Diffraction (μ-XRD^2)^ and optical microscopy in polarising light suggested that the samples were produced from the calcination of naturally abundant limestone near Tell el-Burak mixed with ceramic sherds whose composition suggests a parallel with the fabrics (especially 1A) that characterises the amphorae found in Tell el-Burak^16^. While petrographic analyses of plaster from sites in the Southern Levant are still limited, macroscopic descriptions indicate that shell was commonly used as an aggregate. Moreover, this practice is well-attested in the Aegean and Eastern Mediterranean during the Bronze Age^17^. This suggests that Tell el-Burak followed a distinct plaster technology, differing from the more widespread tradition observed in the region^15^.

The addition of crushed ceramic surely enhanced the physical and mechanical properties of plaster by increasing its stiffness and reducing shrinkage cracking around the aggregates^8,9,18–20^. However, it was concluded that a more detailed archaeometric investigation would be necessary to assess whether the addition of broken pottery sherds was aimed at the production of a hydraulic plaster. In this article, by combining an integrated programme of archaeometric analysis, we demonstrate that the plaster installations at the site are a clear example of hydraulic mortars, and we discuss the implication of this discovery in light of the technological advancements occurring at the time as well as the use of local resources role of Tell el-Burak in the regional economy.

## Archaeological background

**Fig. 1:**
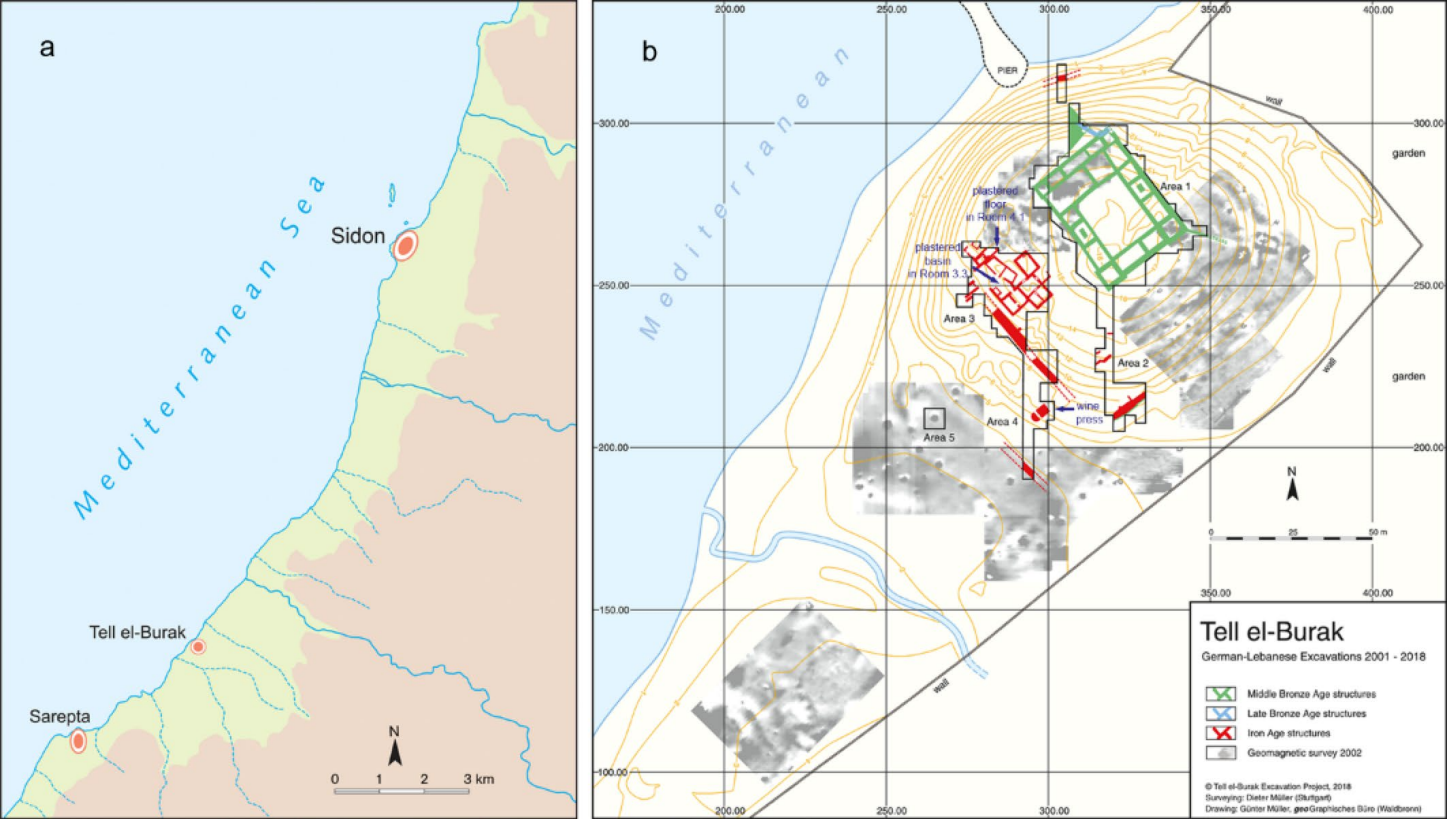
Illustrations related to the geographical and archaeological context of Tell el-Burak: (**a**) Map of the central Levant with the position of Tell el-Burak. (**b**) Plan of the settlement at Tell el-Burak, showing the excavated areas. Images courtesy of the Tell el-Burak Archaeological Project and generated using FreeHand 10. (https://www.adobe.com/mena_en/products/freehand/).

The coastal site of Tell el-Burak, which lies 9 km south of Sidon in southern Lebanon (Fig. 1), was intermittently settled from the Middle Bronze Age I (c. 1900–1700 BCE) to the Mamluk-Ottoman period (c. 13th–17th/18th centuries CE)^21–24^. Its main occupation period dates between approximately 725 and 350 BCE.

Evidence suggests the site functioned as a node in a wider agricultural system, perhaps managed by the nearby polity of Sidon which aimed to harness the favourable environmental conditions of the area. Tell el-Burak provides a detailed snapshot of how the Phoenicians carried out agricultural activities during the Late Iron Age and Persian period. It yielded an impressive array of finds, which include botanical, and animal remains, tools, installations and buildings allowing us to illustrate the various stages of agricultural production in southern Phoenicia: harvest, transport, processing, storing, and consumption activities, which were all placed under divine protection to assure a favourable outcome^15,16,25–27^.

**Fig. 2:**
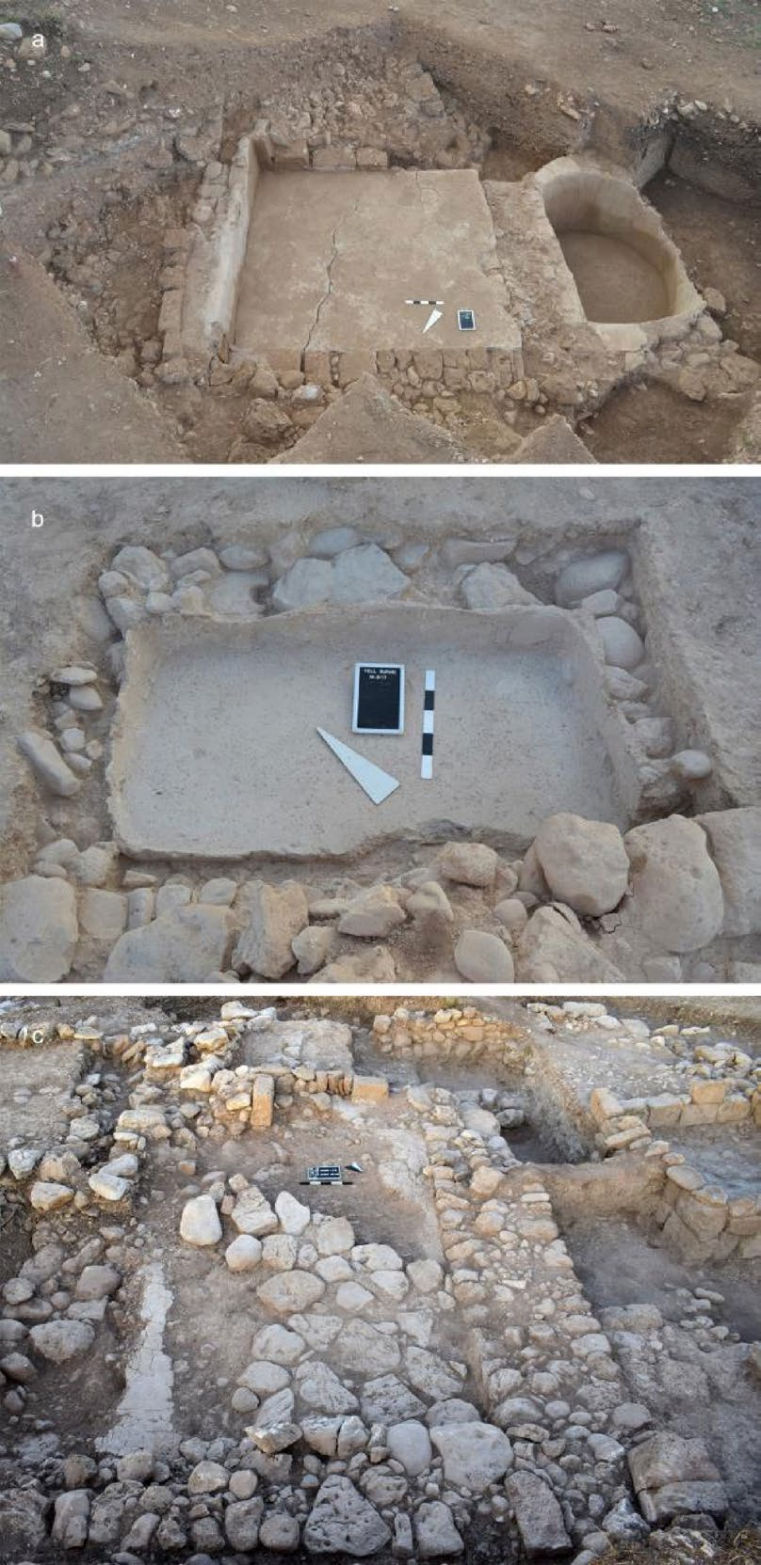
Tell el-Burak, plastered installations: (**a**) Area 4, the wine press, from the west. (**b**) Area 3, the plastered basin in Room 3 of House 3, from the southwest. (**c**) Area 3, the plastered floor in Room 1 of “House 4”, from the northeast (courtesy of the Tell el-Burak Archaeological Project).

The main activity at the site was processing of agricultural products. For this reason, the installations were designed to facilitate the execution of the various operations without causing significant loss of the final product, which mostly consisted of wine and olive oil at Tell el-Burak. Three different plastered installations have been uncovered on site (Fig. 2). The most remarkable is a large and well-preserved wine press (c. 725/700-600 BCE) on the southern slope of the tell (i.e., Area 4). It consisted of a rectangular treading basin (c. 3.20 × 3.50m) where grapes were trodden, and from where the must originally flowed—through a connecting channel—into a (approximately) 4500 litre semi-circular vat (c. 2.50 × 1.95m) where the grape juice was collected and underwent the first fermentation.

The two other structures are located on top of the site (i.e., Area 3), where the remains of four buildings (i.e., Houses 1–4), and a few cultic installations have been uncovered within a walled area. The remains of a basin-like plastered installation (c. 700-600 BCE) were identified underneath the courtyard floor of House 3. Furthermore, House 4—an area with multiple installations, west of House 3—had a rectangular room with remains of a plaster floor and a plastered drainage channel in its southern wall that reached the adjacent room on a lower level (c. 700-600 BCE). How these two other plastered structures were used remains unclear. However, archaeometric analyses have demonstrated that all three facilities had the same plaster composition and technology, comprising a mix of lime and ceramic fragments^15^.

### Analytical approach and materials

To accurately assess the hydraulic properties and compositional complexity of historic mortars, a multidisciplinary analytical approach is essential. This involves optical microscopy, mineralogical, microchemical, and organic residue analyses. Optical microscopy (OM) in polarised light and scanning electron microscopy with energy eispersive spectroscopy (SEM-EDS) provide microstructural and compositional data useful for characterising raw materials, evaluating pozzolanic activity, and detecting early signs of hydraulic phase formation. X-ray powder diffraction (XRPD) is effective for identifying crystalline hydraulic phases, when present, while thermogravimetric analysis (TGA) plays a key role in detecting and quantifying hydrated compounds within the binder, further confirming hydraulic behaviour^2,28^.

To this end, an integrated programme of analyses has been applied to a selection of samples from the above-mentioned plastered installations (Table 1). These specimens were selected based on the existing preliminary petrographic results^15^ and represent the compositional variability observed in the plaster during our previous study.

**Table 1:** List of plaster samples from Tell el-Burak analysed with OM in polarised light, XRPD, SEM–EDS, and TGA.

Sample	Season	Square	Context	OM	XRPD	SEM–EDS	Thermogravimetry
SA1	2018	27/26	House 4, Room1	X	X	X	X
SA2	2018	27/26	House 4, Room 1	X			
SA3	2018	28/25	House 3, Room 3	X	X	X	X
SA4	2018	28/25	House 3, Room 3	X		X	
SA5	2018	28/25	House 3, Room 3	X			
SA6	2018	30/21	Wine press, treading basin	X		X	
SA7	2018	30/21	Wine press, treading basin	X	X		
SA8	2018	29/21	Wine press, receiving vat	X	X	X	X
SA9	2018	29/21	Wine press, receiving vat	X	X		X
SA10	2018	29/21	Wine press, receiving vat	X	X	X	
SA11	2018	30/21	Wine press, treading basin	X			
SA12	2018	30/21	Wine press, treading basin	X			
SA13	2018	30/21	Wine press, treading basin	X			
SA14	2018	30/21	Wine press, treading basin	X			
SA15	2018	30/21	Wine press, treading basin	X			
SA16	2015	29/21	Wine press, receiving vat	X	X		
SA17	2015	29/21	Wine press, receiving vat	X			
SA18	2015	29/21	Wine press, receiving vat	X	X		X

Additionally, 2 samples of plaster and 1 soil control sample were analysed via organic residue analysis (ORA). Organic Residue Analysis aids in identifying natural additives—such as oils, resins, proteins or polysaccharides—that may have been intentionally introduced to influence setting dynamics, durability or water resistance, and can help verify the presence of organic components contributing to hydraulic performance^29^. The 2 plaster samples were taken from the treading floor of the winery, while the soil control was taken from the proximity of the wine press. A sterile scalpel was used for sampling and the plaster fragments were mechanically cleaned before extraction.

Finally, eighteen samples of ceramic sherds found in Tell el-Burak (Table 2) previously characterised via OM in polarised light^16^ were selected to study their mineralogical composition and firing temperatures in more detail. These samples exemplify the most significant ceramic fabrics recognised at the site and therefore most likely represent the type of ceramic added as aggregates in the lime plaster as previously hypothesised^15^.

**Table 2:** List of ceramic sherd samples from Tell el-Burak analysed using OM in polarised light and XRPD.

Sample	Inventory number	Vessel type	Vessel group	Phase	Petrofabric fabrics	Provenance
CR1	2923-019-0363	Amphora	A-01E	E	1C	Southern Lebanon
CR2	2923-019-0511	Mortarium	Unclassified	E	Unclassified	Cyprus
CR3	2923-019-0156	Large bowl	LB-02	E	1C	Southern Lebanon
CR4	2924-0236-0009	Amphora	A-02A	D	1A	Southern Lebanon
CR5	2923-043-0039	Cooking pot	CP-01 K	E	Unclassified	
CR6	2923-019-1108	Amphora	A-01C	E	1C	Southern Lebanon
CR7	2923-019-0280	Large bowl	LB-14	E	1C	Southern Lebanon
CR8	2923-019-0393	Large bowl	LB-04	E	1C	Southern Lebanon
CR9	2923-019-1075	Amphora	A-01E	E	1C	Southern Lebanon
CR10	2924-236-0026	Amphora	A-02A	D	1A	Southern Lebanon
CR11	2924-282-0026	Large bowl	LB-04	E	1C	Southern Lebanon
CR12	2824-030-0341	Bowl	B-06	D/E	1B	Southern Lebanon
CR13	2923-043-0035	Bowl	B	E	Unclassified	Southern Lebanon
CR14	2923-019-0098	Bowl	B-06	E	Unclassified	Cyprus
CR15	2924-197-0294	Amphora	A-01G	D	1A	Southern Lebanon
CR16	2924-236-0022	Amphora	A-02A	D	1A	Southern Lebanon
CR17	2924-019-0714	Amphora	A-01F	E	Unclassified	
CR18	2924-274-0016	Amphora	A-01G	D	1A	Southern Lebanon

## Methods

### XRPD

The mineralogical bulk composition of mortars/plasters was obtained by analysing a selection of eight powdered samples (Table 1) at the Institute of Heritage Sciences, CNR of Sesto Fiorentino (Italy). The instrument used was a X’Pert Pro PANalytical diffractometer equipped with an X’Celerator detector with a Cu X-ray tube (λ = 1.54 Å) and a Ni-filtered Cu–Kα radiation source. The X-ray tube was operated at 40 kV and 30 mA. The diffraction patterns were collected from 3 to 70° 2θ with a step size of 0.02° and total time per pattern of 16 min 27 s. A zero-background sample stage was used, the analytical configuration included a 1/4° divergence slit, 1/2° antiscatter slit, a 15 mm beam mask.

Eighteen ceramic sherds (Table 2) were analysed at the Durham Archaeomaterials Research Centre (United Kingdom). The instrument used was a Panalytical Aeris diffractometer equipped with a PIXcel3D solid-state detector with a Cu X-ray tube (λ = 1.54 Å) and a Ni-filtered Cu–Kα radiation source. The X-ray tube was operated at 40 kV and 15 mA. Measurements were taken from 5 to 70° 2θ at a step size of approximately 0.0054° at 39.5 s per step for a time per pattern of 21 minutes 57 s. The powdered sample was placed in a circular sample holder with a diameter of 32 mm and a depth of 3 mm. A nickel-beta filter was used on the incident side, along with 0.04 rad soller-slits inserted on both the incident and detector side of the beam. The analytical configuration also included a 1/4° divergence slit, a 23 mm beam mask, a beam knife in the ‘hi’ position, and a 9 mm antiscatter slit. The results from both instruments were analysed using the ‘HighScore’ proprietary software package (version 3.0d(3.0.4), Panalytical B.V.; https://www.malvernpanalytical.com/en/products/category/software/x-ray-diffraction-software/highscore) in conjunction with the International Centre for Diffraction ‘Minerals’ Database (ICDD).

### OM in polarised light and SEM–EDS

As mentioned above, the preliminary minero-petrographic study of samples from Tell el-Burak focused on establishing the main compositional characteristic of the samples^15^. In this paper, further OM in polarised light observations on eighteen samples combined with SEM analysis on six samples were employed (Table 1) to have a more detailed identification of the raw materials used to produce the plaster. This study, through the application of these two techniques also aimed to investigate in greater depth the matrix characteristics of the ceramic aggregates, such as porosity and optical activity, as well as the presence of reaction rims that might indicate the hydraulicity nature of the material under study^30,31^. The addition of crushed ceramic or bricks normally creates reaction rims on the side of the carbonate binder, which appear darker than the centre of the ceramic fragment in BSE imaging^32^, and are characterised by a decrease in the silicon content that becomes more pronounced near the interface with the calcium-rich binder. The hydraulicity index (HI) was calculated based on chemical data obtained via SEM–EDS analysis of the lime lumps, binder and reaction rims^33^.

The petrographic analysis on thin sections (30 µm thickness) was carried out in transmitted light using an optical microscope (OM). For mortars, this approach allowed the precise identification of the binder, aggregates, lumps and inorganic additives and admixtures^30,32^. A Zeiss Axioscope A.1 microscope with a magnification of 25x to 400x, equipped with a camera for image acquisition and special software (AxioVision, Carl Zeiss Microscopy, LLC, USA–version AxioVS40 V 4.8.2.0, Carl Zeiss Microscopy; https://carl-zeiss-axiovision-rel.software.informer.com) for image processing and measurement of the most important material properties was used for the investigation.

SEM-EDS analyses were carried out to perform microstructural and semi-quantitative chemical analyses of the binder and aggregates. A SEM-EDS (ZEISS EVO MA 15) with W filament equipped with analytical system in dispersion of energy EDS/SDD, Oxford Ultimax 40 (40 mm^2^ with resolution 127 eV @5.9keV), with a magnification of 100x to 100kx was used. The operative conditions included an acceleration potential of 15 kV, 500 pA beam current, a working distance between 9 and 8.5 mm, 20s live time as acquisition rate useful to archive at least 600.000 cts, on Co standard, process time 4 for point analyses, 500 µs pixel dwell time for maps acquisition with 1024×768 pixel resolution. The programme used for microanalysis was an Aztec 5.0 SP1 software that employs the XPP matrix correction scheme developed by Pouchou and Pichoir in 1991^34^.

### TGA

A selection of samples (Table 1) was disaggregated using a porcelain pestle, and the fraction that passed through a 63 µm sieve (ISO R 565 Series) was considered the binder-enriched portion of the mortar sample. TGA was used to evaluate the presence and the amount of particular volatile compounds (essentially H_2_O, CO_2_) in these portions. TGA was conducted in the range 110–1000 °C on approximately 4–5 mg of sample, and dried (silica gel as drying agent) at room temperature for at least a week, under the following experimental conditions: open alumina crucibles, heating rate of 10 °C/min, and 20 ml/min nitrogen gas flow. TGA was used for classifying the studied samples as hydraulic or non-hydraulic mortars^35–37^. Three measurements were performed for each sample.

### ORA

All glassware was sterilised prior to use (500°C, 8 hours), and HPLC Grade solvents were used. Solvent extraction followed published protocols^38^, and subsequently the application of a short-chain carboxylic compound extraction following Garnier and Valamoti (2016)^39^ was carried out. A detailed description of the methodology applied for solvent extraction can be found in the Supporting Information (SI 1).

Gas Chromatography (GC) and GC-Mass Spectrometry (GC-MS) analyses were carried out using an Agilent Technologies 7890 GC System series chromatograph including an Agilent Technologies Capillary Flow-Technology Three-Way Splitter coupled to an Agilent Technologies 5977A Mass Spectrometry Detector (MSD) and Flame Ionisation Detector (FID). Splitless injection was performed using a GERSTEL Multipurpose Sampler and GERSTEL Cold Injection System (CIS) 4. The GC was fitted with an Agilent J&W DB-5HT-column (15m × 0.32mm i.d.; 0.1μm film thickness) and the eluent divided into two equal parts using 0.18mm non-coated, deactivated silica capillary columns (0.66m splitter-column to the FID/ 1.52m splitter-column to the MSD) with the Three-Way Splitter Kit. The inlet temperature was ramped from 30 to 240 °C at 12 °C s^−1^ (5 min. isothermic hold) and then to 350 °C at 10 °C s^−1^ (10 min. isothermic hold). The oven temperature was initially set at 40 °C (1 min. isothermic hold), ramped to 100°C at 15°C min⁻^1^, and then to 240 °C at 6 °C min^−1^, and finally increased to 350 °C at 10 °C min^−1^ (20 min. isothermic hold). Helium was used as the carrier gas. The split/splitless injection system was operated in splitless mode with a purge flow of 3.0 ml min^−1^ and a constant pressure at the head of the column of 8.4435 psi. The FID temperature, transfer line and ion source were held at 300 °C. Electron Ionisataion (EI) spectra were obtained at 70eV with a full scan from m/z 50 to 950. The butylated extracts were run under the same conditions, with the following modifications. The oven temperature was ramped from 50 °C (held isothermally for 1 min) to 100 °C at 15°C min^−1^, then to 240 °C at 4 °C min^−1^ and increased to 350°C at 20 °C min^−1^ (held isothermally for 7 min). The temperature of the FID, ion source and transfer line were 340 °C, 230 °C and 300 °C, respectively. Data was acquired using an MSD ChemStation F.01.01.2317. Mass spectra were matched against authentic standards (saturated and unsaturated triglycerides, fatty acids, alkanes, and short-chain carboxylic compound), published literature and the National Institute of Standards and Technology (NIST) library, 2014 edition.

## Results

### XRPD

Calcite, quartz were identified in all the plaster samples. The main peaks of gehlenite (d 2.84 Å–31.42° 2θ), and weak peaks of cristobalite (4.04 Å–21.99 °2θ) as well as the mullite (3.40 Å–26.18 °2θ) were detected in all the samples apart from SA10. Some samples also show very weak reflections that could be connected to K-feldspars, while a shoulder at 2.99 Å–29.91°2θ might suggest the presence of diopside in a few samples (Table 3 and SI 2).

**Table 3:** Results of XRPD analysis carried out on a selection of plaster samples from Tell el-Burak.

Sample	Mineralogical composition*
SA1	Calcite, quartz, gehlenite, cristobalite, mullite
SA3	Calcite, quartz, gehlenite, cristobalite, mullite
SA7	Calcite, quartz, K-feldspars, gehlenite, cristobalite, mullite, diopside?
SA8	Calcite, quartz, K-feldspars, gehlenite, cristobalite, mullite, diopside?
SA9	Calcite, quartz, K- feldspars, gehlenite, cristobalite, mullite, diopside
SA10	Calcite, quartz
SA16	Calcite, quartz, K-feldspars, gehlenite, cristobalite, mullite, diopside
SA18	Calcite, quartz, K-feldspars, gehlenite, cristobalite, mullite, diopside

*in order of abundance.

Calcite, quartz as well as K-feldspars (Table 4 and SI 2) were also identified in all the ceramic sherd samples analysed. However, there is no evidence for gehlenite, cristobalite, mullite and diopside, while all the samples show the main peak of muscovite/illite at d 4.48 Å–19.82° 2θ. Finally, few samples might contain hematite as a weak peak at d 2.70 Å–33.15° 2θ.

**Table 4:** Results of XRPD analysis carried out on a selection of ceramic sherd samples from Tell el-Burak.

Sample	Inventory number	Mineralogical composition*
CR1	2923-019-0363	Calcite, quartz, muscovite/illite, hematite, K-feldspars
CR2	2923-019-0511	Calcite, quartz, muscovite/illite, hematite, K-feldspars, amphibole
CR3	2923-019-0156	Calcite, quartz, muscovite/illite, hematite, K-feldspars
CR4	2924-0236-0009	Calcite, quartz, illite/muscovite, K-feldspars
CR5	2923-043-0039	Calcite, quartz, muscovite/illite, K-feldspars
CR6	2923-019-1108	Calcite, quartz, muscovite/illite, hematite?, K-feldspars
CR7	2923-019-0280	Calcite, quartz, illite/muscovite, hematite?, K-feldspars
CR8	2923-019-0393	Calcite, quartz, illite/muscovite, K-feldspars
CR9	2923-019-1075	Calcite, quartz, illite/muscovite, K-feldspars
CR10	2924-236-0026	Calcite, quartz, illite/muscovite, K-feldspars
CR11	2924-282-0026	Calcite, quartz, illite/muscovite, K-feldspars
CR12	2824-030-0341	Calcite, quartz, muscovite/illite, K-feldspars
CR13	2923-043-0035	Calcite, quartz, illite/muscovite, K-feldspars
CR14	2923-019-0098	Calcite, quartz, plagioclase, muscovite/illite, amphibole
CR15	2924-197-0294	Calcite, quartz, muscovite/illite, K-feldspars
CR16	2924-236-0022	Calcite, quartz, illite/muscovite, K-feldspars
CR17	2924-019-0714	Calcite, quartz, muscovite/illite, K-feldspars
CR18	2924-274-0016	Calcite, quartz, illite/muscovite, K-feldspars

*in order of abundance.

### OM in polarised light and SEM–EDS

**Fig. 3:**
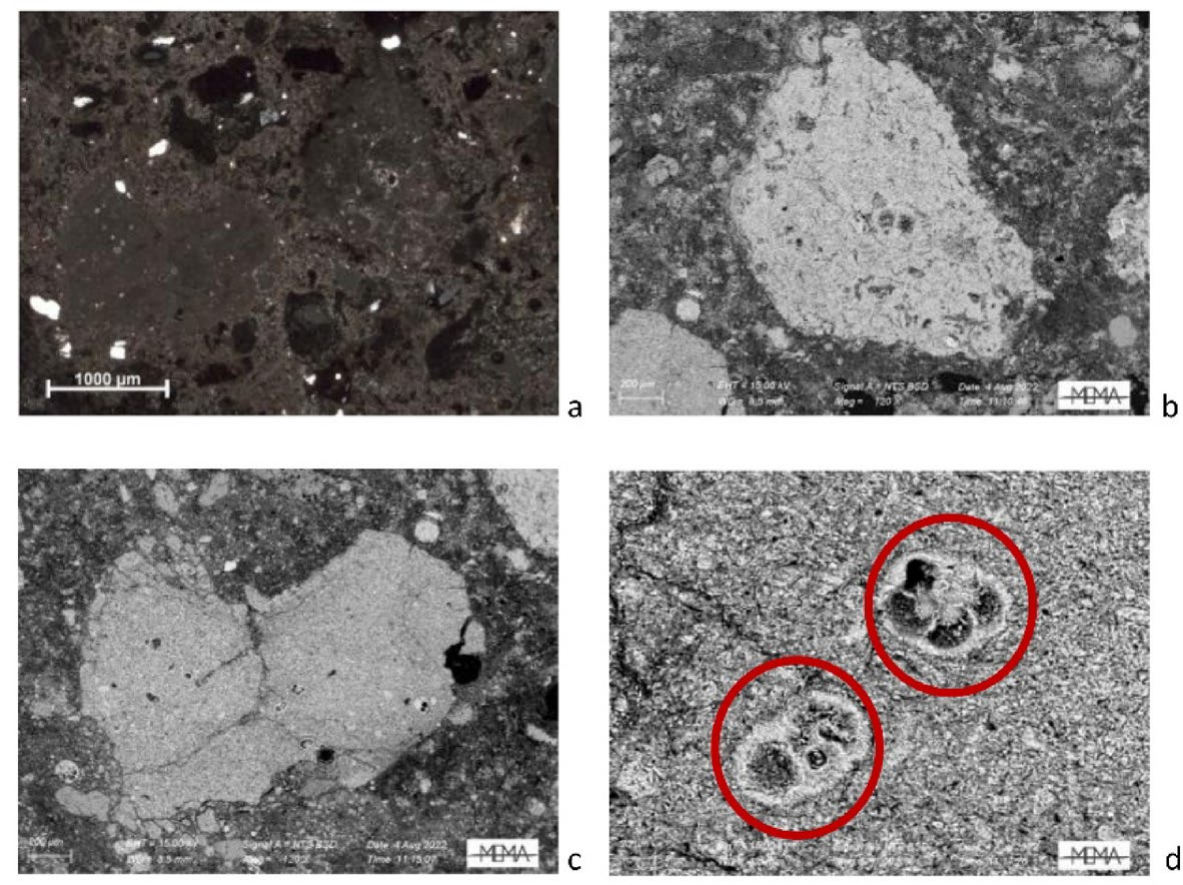
Thin section microphotographs of two thermally altered limestone fragments in sample SA3: (**a**) Polarising microscope, XP; (**b**, **c**) BSE images at high magnification; (**d**) BSE image showing a particular section of limestone with visible micro-fossiliferous content.

Concerning the stone used for binder production, two fragments of thermally altered carbonate were identified in SA3 via OM analysis (Fig. 3a) and observed at higher magnification in SEM-BSE (Fig. 3b, c), revealing an almost homogeneous structure with few quartz and bioclast inclusions. Well-preserved bioclasts, identified as foraminifera, were detected too (Fig. 3d). In addition, lime lumps are widespread in all examined samples.

SEM-EDS micro-chemical analyses were performed on limestone, lime lumps, plaster binder, reaction rims between ceramic fragments and binder, and ceramic fragments. The full results are presented in Table 1 (in SI 3) and Figs. 1 and 2 (in SI 3). Analyses of points and areas on lime lumps and limestone show high CaO concentrations ranging from 70.9 to 96.3%, with no evidence of natural hydraulicity in the lime lumps (HI < 0.01). Silica is confined to small, unreactive quartz grains. Low levels of MgO were detected (< 1.6%) in both lime lumps and limestone.

**Fig. 4:**
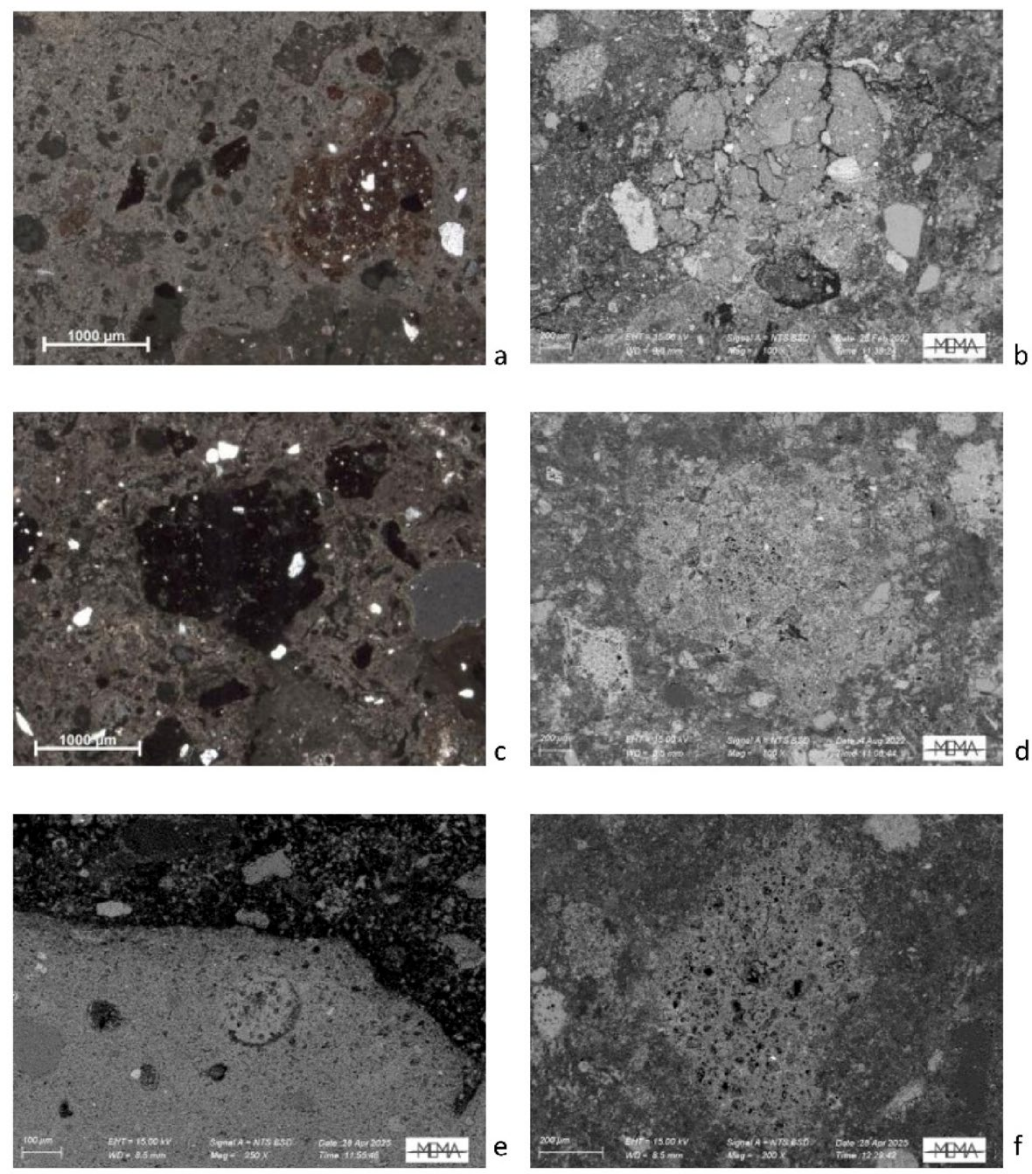
Thin section microphotographs showing two different types of ceramic aggregates: (**a**) Type 1, polarising microscope, XP; (**b**) Type 1, BSE image at high magnification; (**c**) Type 2, polarising microscope, XP; (**d**) Type 2, BSE image at high magnification; (**e**) Type 1, BSE image at high magnification; (**f**) Type 2 with bloating pores, BSE image at high magnification.

Concerning ceramic aggregates, the former minero-petrographic study indicated that the plaster was obtained by mixing lime-based micritic binder and abundant fragments of ceramic with other aggregates^15^. Quartz, shells, microfossils, opaque minerals, cherts and organic inclusions (i.e. charcoal) as well as rare feldspars and fragments of sedimentary rocks were identified. The new combined OM and SEM-EDS observations show that the fragments exhibit a heterogeneous matrix in terms of porosity and optical activity. However, they can be broadly categorised into two types: ceramic fragments with a fine-grained matrix, displaying varying degrees of optical activity, red to orange in OM, high porosity and with few sub-rounded quartz grains as inclusion (type 1, Fig. 4a, b, e); and ceramic fragments with an isotropic matrix, dark grey to black in OM, low porosity, though occasionally showing bloating pores, and with rare quartz grains as inclusion (type 2, Fig. 4c, d, f).

**Fig. 5:**
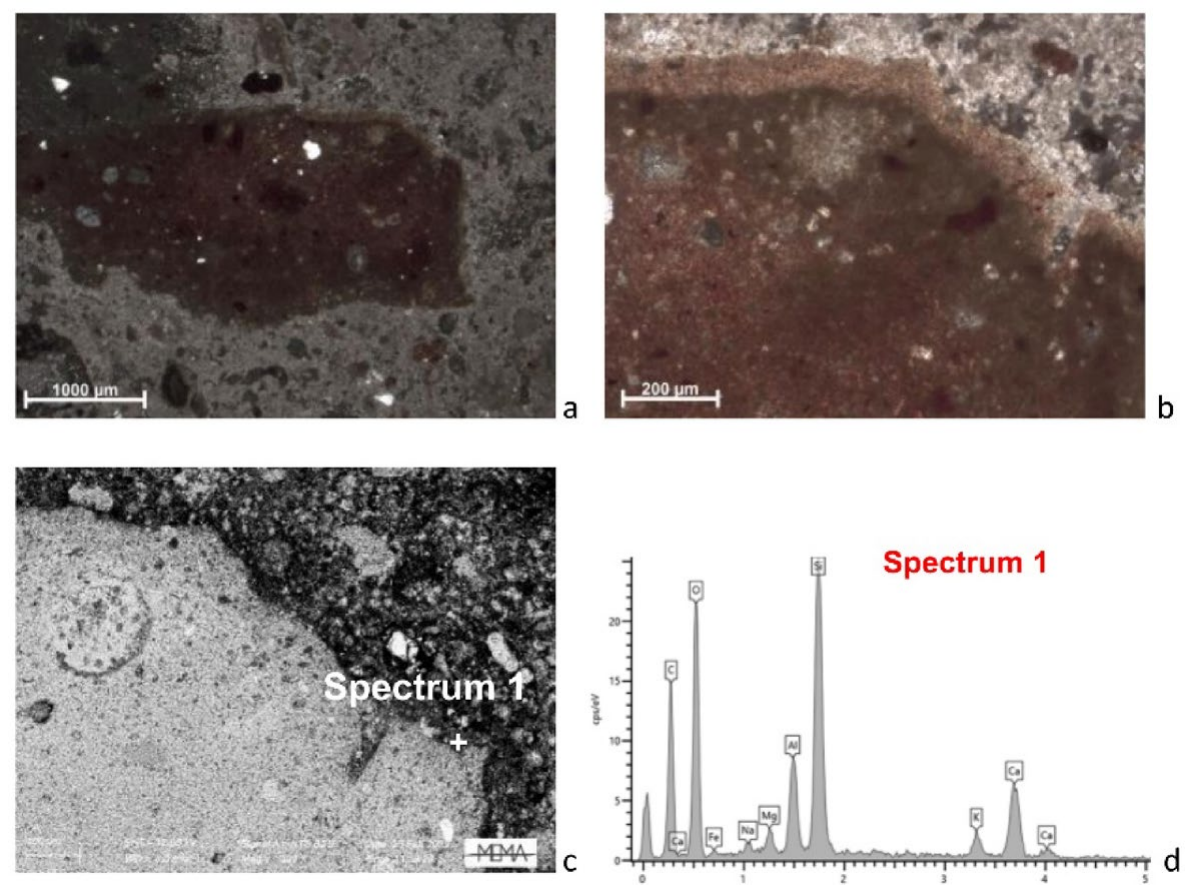
SEM–EDS analysis of reaction rims (Type 1): (**a**) and (**b**) Reaction rim between a ceramic fragment and a calcitic lime in sample SA1 at different magnifications, polarising microscope, XP; (**c**) BSE image of the reaction rim; (**d**) EDS spectrum of the reaction rim.

**Fig. 6:**
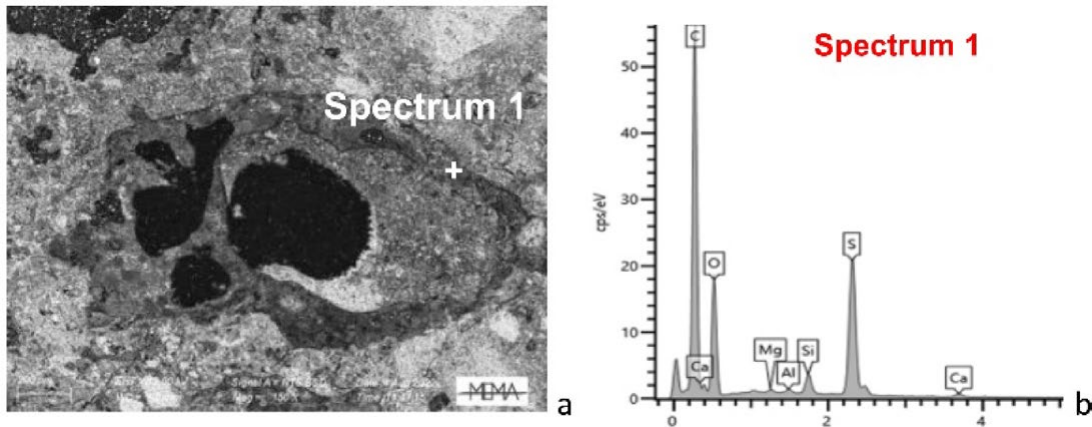
SEM–EDS analysis of sample SA6: (**a**) BSE image of a highly porous area; (**b**) EDS spectrum of the area.

In some samples (i.e. SA1, SA3, SA8, SA10) reaction rims between the ceramic fragments and lime were also observed via OM, above all in the case of presence of type 1 ceramic fragments (Fig. 5a, b). The same areas observed in SEM BSE mode appear darker (Fig. 5c) marked by the development of silica-alumina calcium new phases as confirmed by the collected EDS spectrum (Fig. 5d). The calculated Hydraulicity Index (HI) in the reaction rims ranges from 0.13 (sample SA3) to 0.80 (sample SA1), and from 0.02 (sample SA4) to 0.62 (sample SA10) in the binder, indicating high heterogeneity—even within the reaction rim of the same ceramic aggregate—and suggesting that the samples exhibit weak to moderate hydraulicity (Table 1, in SI 3). However, the presence of reaction rims confirms that the observed hydraulicity is attributable to the addition of ceramic fragments (Fig. 5; Fig. 1, in SI 3). Finally, in porous areas of the binder (Fig. 6a), EDS analyses revealed the systematic presence of S, as recorded in the spectrum (Fig. 6b).

### TGA

**Fig. 7:**
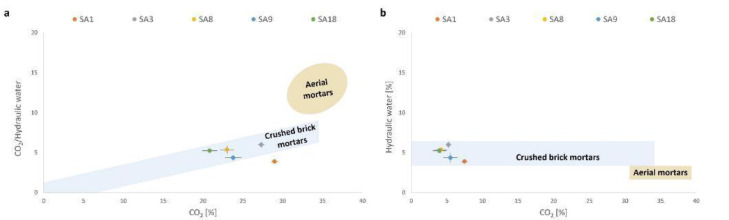
Results of thermogravimetry analysis: (**a**) Plot of weight loss attributed to CO_2_/hydraulic water vs % CO_2_; (**b**) Plot % of weight loss attributed to hydraulic water vs CO_2_.

The weight loss in the 200–600 °C temperature range observed in TGA analysis (Fig. 7) indicates the dehydration of the aluminosilicates that is connected to the presence of hydraulic components; in the range 600–900 °C temperature, the decomposition of CO_2_ is registered.

The amount of hydraulic water in aerial mortars is lower than 3%, while higher than 3% in the hydraulic mortars^37^. The amount of CO_2_ in the sample comes from the calcium hydroxide carbonation process and the contribution of calcium hydrated silico-aluminates. The latter react slowly with CO_2_ forming CaCO_3_ and separating silica and alumina in the amorphous state. This parameter makes it possible to distinguish mortars with aerial lime characterised by the loss of CO_2_ > 30% compared to mortars with hydraulic lime with values < 30%^35,36^.

All the analysed samples have a percentage of water bound to hydraulic components higher than 3%, the values ranging from 3.74 to 7.52%. The % CO_2_ varies between 19.80% and 30.00%. All the analysed samples, thus, fall in the cluster of hydraulic mortar obtained by addition of crushed ceramics (crushed bricks mortars as defined in^37,40^), concentrated in the middle of the plot, and well distinguished from typical lime mortars at the upper right part of the figure (Fig. 7a, b) This thermogravimetric behaviour of crushed brick mortars is attributed to water bound to several calcium aluminium silicate hydrates.

### ORA

**Fig. 8:**
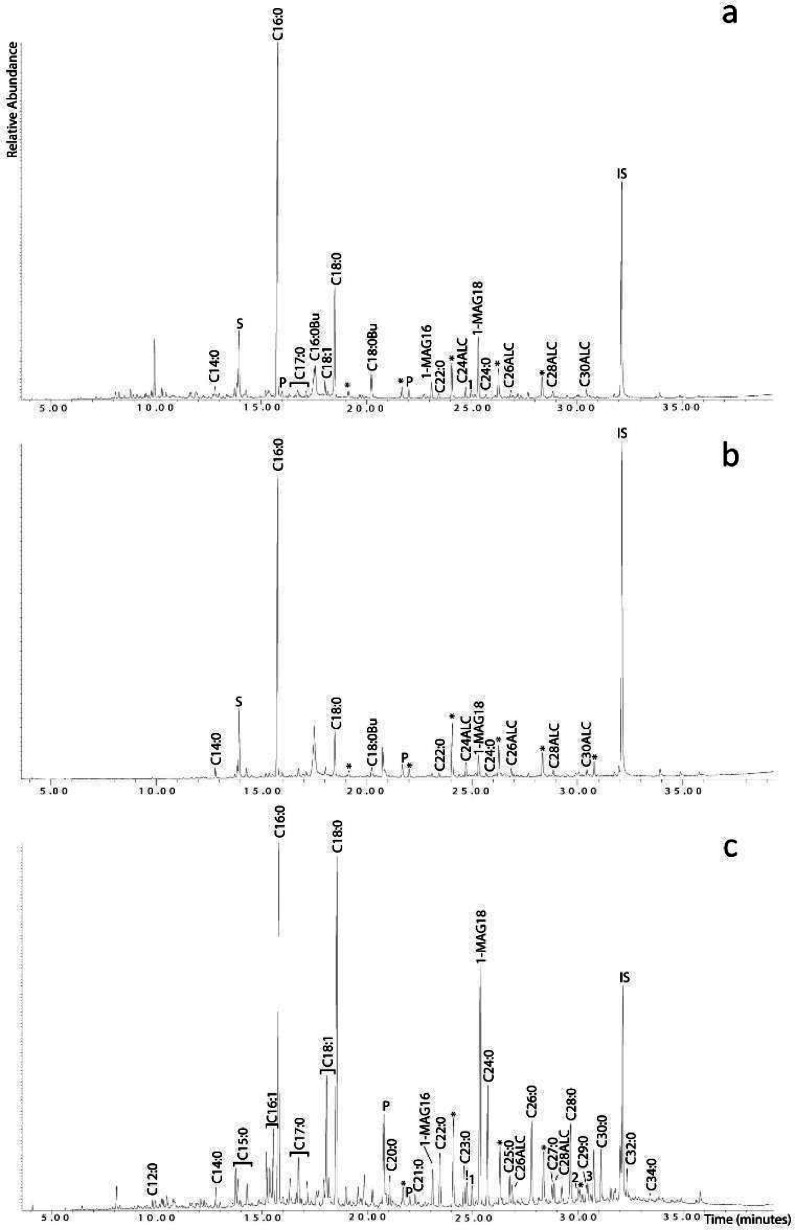
Partial total ion chromatogram of the two plaster samples taken from the treading floor of the winery: (**a**) TLB-5P; (**b**) TLB-6P; (**c**) the soil control sample. Sulphur can be seen to be present in the plaster samples but absent in the soil control sample tested [Cx:y: Fatty Acids showing the number of carbon atoms (x) and the number of unsaturations (y), Bu: Butylated Fatty Acids; ALC: Alcohols showing carbon number; *: Alkanes; !: C_24_ Alcohol; 1: 2-monostearin; 2: β-sitosterol; 3: C_30_ Alcohol; S: Sulphur, P: Phthalates; IS: Internal standards; MAG: Monoacylglycerols].

The two plaster samples (Fig. 8) taken from the treading floor of the wine press contained relatively few organic compounds, namely saturated fatty acids (C_14:0_–C_24:0_), unsaturated fatty acids (C_18:1_), branched fatty acids (C_17:0_), *n*-alcohols (C_24_–C_30_), monoacylglycerols (1-monopalmitin, 2-monostearin, and 1-monostearin), and a series of *n*-alkanes. Of interest was the presence of sulphur, which was observed in both plaster samples but not in the soil control sample.

The soil control sample was taken from the soil covering the treading floor of the wine press and was analysed to test for exogenous contamination. It comprised saturated (C_12:0_–C_34:0_), unsaturated (C_16:1_ and C_18:1_), odd-chained (C_15:0_–C_29:0_) and branched (C_15:0_ and C_17:0_) fatty acids, monoaclyglycerols showing the onset of triacylglycerol hydrolysis, *n*-alcohols (C_24_–C_30_), a series of *n*-alkanes, and a single phytosterol (β-sitosterol). The lipid profile obtained shows a characteristic soil lipid distribution reflecting animal and plant detritus^41^. The absence of sulphur is noted and confirms its presence solely within the plaster samples.

## Discussions

### Raw materials and technology

Previous archaeometric analyses carried out on the plaster samples from the three installations in Tell el-Burak suggested the possible existence of a consistent technological tradition of lime plaster production at the site, that made use of fragments of ceramic as the main aggregate^15^. The new set of analyses now gives a deeper understanding of the compositional and technological characteristics of the plaster installations from Tell el-Burak.

Both OM in polarising light and SEM allow the identification of thermally altered carbonate fragments deriving from the original limestone used for the lime production. This limestone contains coral fragments, small benthic and planktonic foraminifera and, rarely, large benthic foraminifera^15^. Such microfossils occur regularly in Eocene and Miocene chalks found in the area spanning from the north of Sidon to the south of Tyre with sources available near Tell el-Burak^16,42^.

The use of gypsum to produce binder^43^ that might be suggested by the presence of sulphur, detected both via SEM and ORA analyses, can be excluded as this mineral or phases deriving from its thermal alteration, such as hemihydrate and anhydrite, were not identified with any of the analyses carried out on the studied materials. The presence of sulphur instead might be connected to the use of these installations for wine production, and/or perhaps the presence of ceramic inclusions as plaster aggregates which may derive from wine amphorae^16^, by far the most common ceramic type at the site. Sulphur has previously been reported in other studies in relation to wine consumption from ceramic vessels (see e.g.^44–47^), and various hypotheses have been put forward to explain its presence, including its use as a surface treatment, antifungal agent and as a wine preservative^45,47,48^.

The OM in polarising light and SEM analysis also confirmed the addition of different types of ceramic fragments as aggregates. The addition of the latter could enhance the mechanical properties of plaster, by increasing its stiffness and reducing shrinkage-related cracking around the aggregates, but it could be also connected to the production of a high-quality hydraulic lime plaster. Notably, the SEM-EDS and TGA analyses indicate that the addition of ceramic fragments confers hydraulic behaviour to plasters, highlighting the presence of reaction rims between binder and aggregate, and the amount of calcium silico-aluminate phases formed during setting and hardening of plasters.

Therefore, we can conclude that the addition of ceramic fragments into lime plaster was likely aimed at obtaining a hydraulic plaster, improving the physical and mechanical properties of this material.

As discussed earlier, the addition of ceramic fragments as aggregate is an unusual technological choice for the area, as previous studies indicate the preferential use of shell as additives in the production of plaster^15^. It has been also noted that grog is very rarely used as temper in pottery in pre-classical Lebanon^49^ and it is not attested in the ceramic found at Tell el-Burak^16^. These points, taken together with the fact that sand found next to the site was not used as the main aggregate, as well as the evidence of the hydraulicity of the plaster, reinforce the hypothesis that the addition of ceramic fragments into lime was a well-defined technological choice not influenced by environmental constraints^15^.

### Ceramic aggregates

The XRPD analysis carried out on the plaster samples revealed the presence of other minerals, additional to those detected in our previous study via highly resolved μ-XRD^2^ (calcite and quartz) measurements, that focused only on the binder^15^. These include gehlenite, cristobalite, mullite, K-feldspars and probably a weak reflection of diopside. As these phases were not detected in the pure binder, they can be connected to the ceramic fragments added as aggregate into the lime.

Some of these minerals are indeed typically associated with relatively high fired ceramics (e.g.^50,51^). More precisely, gehlenite and diopside normally start to form during the reaction of CaO and the decomposition of clay minerals above 800 °C^50,52,53^. On the other hand, mullite and cristobalite normally nucleate above c. 1000 °C^50^.

As mentioned above, the analysis carried out using OM in polarised light indicates that the matrix of some ceramic aggregates (Type 2) is isotropic—an attribute typically associated with firing temperatures exceeding 800–850 °C^54^. Complementary SEM analysis reveals that these isotropic fragments exhibit extensive to continuous vitrification, and in some cases, the development of bloating pores—features indicative of firing temperatures above 1050 °C, characteristic of overfired ceramics^55^.

Based on these observations, and as previously noted, the ceramic fragments can be broadly categorised into two types, corresponding to general firing temperature ranges: Type 1 fragments, which display an optically active matrix, are consistent with relatively low firing temperatures (below 800–850 °C); and Type 2 fragments, characterised by an isotropic matrix, higher degrees of vitrification, and occasional bloating pores, suggest exposure to higher firing temperatures exceeding 800–850 °C and in some cases likely beyond 1050 °C. However, it is important to emphasise the significant heterogeneity observed both between ceramic aggregates but also within individual ceramic fragments themselves, reflecting a broad spectrum of firing conditions. This variability underscores that the classification into Type 1 and Type 2 is best understood as a general interpretative framework rather than a definitive typology.

The XRPD analysis of the ceramic sherds from a wide range of types contemporary with the installations (phase E and D) do not show the presence of any mineral connected to high firing temperatures. On the contrary, most of the sherds show the presence of calcite and illite which decompose respectively at c. 800 °C and 900 °C^50^ suggesting average firing temperatures below this range.

Although the ceramic fragments found within the plaster are too small for systematic petrographic study, their composition indicated a parallel with the main fabrics (especially fabric 1A) associated with amphorae found at the Tell el-Burak that were likely produced by the long-established community of potters at contemporaneous site of Sarepta 4 km away^16^. The analysis via ceramic thin-section petrography of more than 200 samples of these amphorae, including those of fabric 1A, also indicated that the estimated temperatures were generally below 800–850 °C^16^, a result that correlates with maximum temperature estimated in this study via XRPD.

Overfired sherds (wasters) discarded after production are quite conspicuous in appearance, and, importantly, these have not until now been identified amongst the millions of sherds collected and studied at the extensively excavated and surveyed site of Tell el-Burak, though are reported at the known production site of Sarepta^16^. The evidence, therefore suggests that aggregates were not selected from the abundant discarded ceramic sherds or sands easily obtained around Tell el-Burak, but rather that efforts were made to source waste connected to pottery production or perhaps purpose-prepared ceramic materials for lime plaster production. Regardless, the evidence shows that plaster making was clearly undertaken by people with a specialised knowledge via well-defined modes of production, utilising consistent recipes drawing on specially selected material.

This evidence further supports recent understandings of the organisation of craft and agricultural production/distribution in the Southern Phoenician economy as elite, administered, centralised and specialised which gave those in charge the ability to draw on resources as needed (^16^ and literature therein). Excavations and the analysis of amphorae from Tell el-Burak leave no doubt that wine production was a critically important aspect of the economy and our study reinforces this by showing that even the production of the plastered infrastructure would be undertaken via consistent, specialised, and centralised modes drawing on innovative new technologies.

With the above in mind, the effect and any benefit of using materials containing overfired ceramics as a source of aggregates remains unclear as these are comparatively much less reactive than ceramics fired below 900 °C, to which the plasterers clearly also had access^56–58^. Any advantages in terms of workability, drying or strength would surely have been weighed against the extra effort and fuel required to produce a mix containing specially prepared highly fired materials^56,57^. Therefore, it is likely, the producers were drawing on production waste, a mix of overfired and broken or rejected vessels, rather than intentionally manufacturing ceramics specifically for this use. This waste material likely contained enough sherds fired at a temperature range between 600–900 °C, which reacted well with the lime to achieve the desired hydraulicity.

The presence of ceramic aggregates fired at different range of temperatures is not surprising. Typically, pottery production waste includes a mix of overfired and properly fired sherds rejected for other flaws^59^. The producers may not have had the opportunity to determine the ineffectiveness of adding overfired sherds as they always (whether by choice or circumstance) seemed to draw on similar materials which, in any case, contained sufficient components with pozzolanic properties. This last point is evidenced by the plasters found on three separate installations being similarly composed.

That these materials weren’t sourced locally, as no production wasters were found at Tell el-Burak, and provided no functional advantage over locally available consumption waste materials opens the possibility that the producers also came from elsewhere, perhaps from the nearby production site of Sarepta. The evidence supports an understanding that the knowledge and skills to produce this plaster was not widely disseminated or, perhaps, even constrained, if one accepts the notion that such construction and other production activities took place under elite support and mediation^17^.

## Conclusions

The interdisciplinary study conducted at Tell el-Burak has yielded important insights into the technological advancements and economic strategies involved in lime plaster production during the Iron Age. The research demonstrates that the people operating at Tell el-Burak had the expertise to produce high-quality hydraulic mortars, a technology that was likely critical for constructing durable agricultural infrastructure, including the well-preserved wine press, which reflects the high level of specialisation in agricultural production at the site^15^.

The discovery that ceramic fragments were deliberately added to the lime plaster to enhance its hydraulic properties reveals a sophisticated level of technological innovation consistent with broader Mediterranean practices. This technique, widely diffused in the Greek world and perfected by the Romans, enabled the production of high-quality plaster and mortars through the addition of “pozzolanic material” like ceramics. Notably, the analysis of the studied materials indicates the use of pottery production waste as a source of aggregates with pozzolanic properties. This finding suggests a technologically advanced and sustainable production process that employed recycled ceramic materials. This not only enhanced the plaster’s hydraulic properties but also might have represented an efficient use of available resources, reducing waste from pottery production. This practice might reflect a sophisticated and environmentally conscious approach, contributing to the durability of agricultural infrastructure such as the wine press. This discovery is significant, as it reflects both the use of local resources, such as readily available limestone from the surrounding environment, and an innovative approach to plaster production through the incorporation of ceramic material as aggregate. We propose that this ceramic material was brought to the site from nearby Sarepta, since ceramic production wasters are not found at Tell el-Burak. It is also reasonable to suggest that individuals from Sarepta, possessing specialised knowledge in plaster production, may have deliberately chosen to use familiar and trusted waste materials to ensure consistency and compatibility with their established techniques. By sourcing and transporting selected materials, they could maintain greater control over quality and reduce the risk of failure during preparation. That said, we acknowledge that confirming the identity and origin of these individuals who produced plaster at Tell el-Burak, as well as fully understanding their motivations for this material selection, lies beyond the scope of the present study. A more comprehensive understanding would require broader investigations into regional plaster technologies, research, which, at present, remains limited.

Regardless of this localised context, the findings should also be considered within the broader framework of Phoenician technological exchange and movement across the Mediterranean. Their migrations and far-reaching networks account for their key role in the westward transmission of ideas and technologies from the Levant (famously including the Alphabet). As such, they are highly credible vectors for the spread of new plaster technologies across the Mediterranean and its eventual take up in the Roman world. Admittedly, the evidence for dissemination remains vague, though our study represents an important contribution, and it is hoped future work across the region will complete the picture. Overall, these results contribute to broader discussions on Phoenician knowledge of hydraulic mortar technology and their influence across the Iron Age Mediterranean. This research enhances our understanding of first millennium BCE technology and provides a valuable contribution to the study of ancient construction techniques and their socio-economic contexts around the Mediterranean.

## Electronic supplementary material

Below is the link to the electronic supplementary material.

Supplementary Material 1

Supplementary Material 2

Supplementary Material 3

## Data Availability

All data generated or analysed during this study are included in this published article (and its Supplementary Information files). The additional data (XRPD diffractograms) that support the findings of the study are openly available at https://doi.org/10.7910/DVN/F7MSOB.
