# Supplementary material for: Innovation through recycling in Iron Age plaster technology at Tell el-Burak, Lebanon
Source: Sci Rep. 2025 Jul 7;15:24284. doi: 10.1038/s41598-025-05844-x (PMC12234981; doi:10.1038/s41598-025-05844-x)
Supplement: Supplementary file 1 — Supplementary Material 1 [file 41598_2025_5844_MOESM1_ESM.pdf]

Solvent Extraction Methodology

Solvent extraction was carried out as follows. Prior to extraction, 10µg of tetratriacontane (C34 *n*-alkane) internal standard were added to each of the plaster and soil samples for quantification purposes. A method blank was included to monitor for possible laboratory contamination. Dichloromethane:methanol (5mL, 2:1, *v:v*) was added to each sample, which were then sonicated (15 minutes) and centrifuged (1500 rpm; 10 minutes). The solvent was decanted into clean scintillation vials. The extraction process was repeated twice more, combining the extracts. The extracts were partitioned and then evaporated to dryness under a gentle stream of nitrogen and mild heating. Half of the aliquot was stored, the other half derivatised (*N,O*-bis(trimethylsilyl) triflouracetamide (BSTFA) with 1% trimethylchlorosilane (TMCS; 4µL pyridine; 10µL DCM; 40°C; 30 minutes), and run as trimethylsilylated derivatives. Hexatriacontane (C36 *n*-alkane, 1µg) was added after re-solubilisation with cyclohexane, for quantification purposes. Both the plaster and soil samples were re-extracted following Garnier and Valamoti (2016)[27] to check for the presence of short-chain carboxylic compounds.
