# Supplementary material for: Innovation through recycling in Iron Age plaster technology at Tell el-Burak, Lebanon
Source: Sci Rep. 2025 Jul 7;15:24284. doi: 10.1038/s41598-025-05844-x (PMC12234981; doi:10.1038/s41598-025-05844-x)
Supplement: Supplementary file 2 — Supplementary Material 2 [file 41598_2025_5844_MOESM2_ESM.pdf]

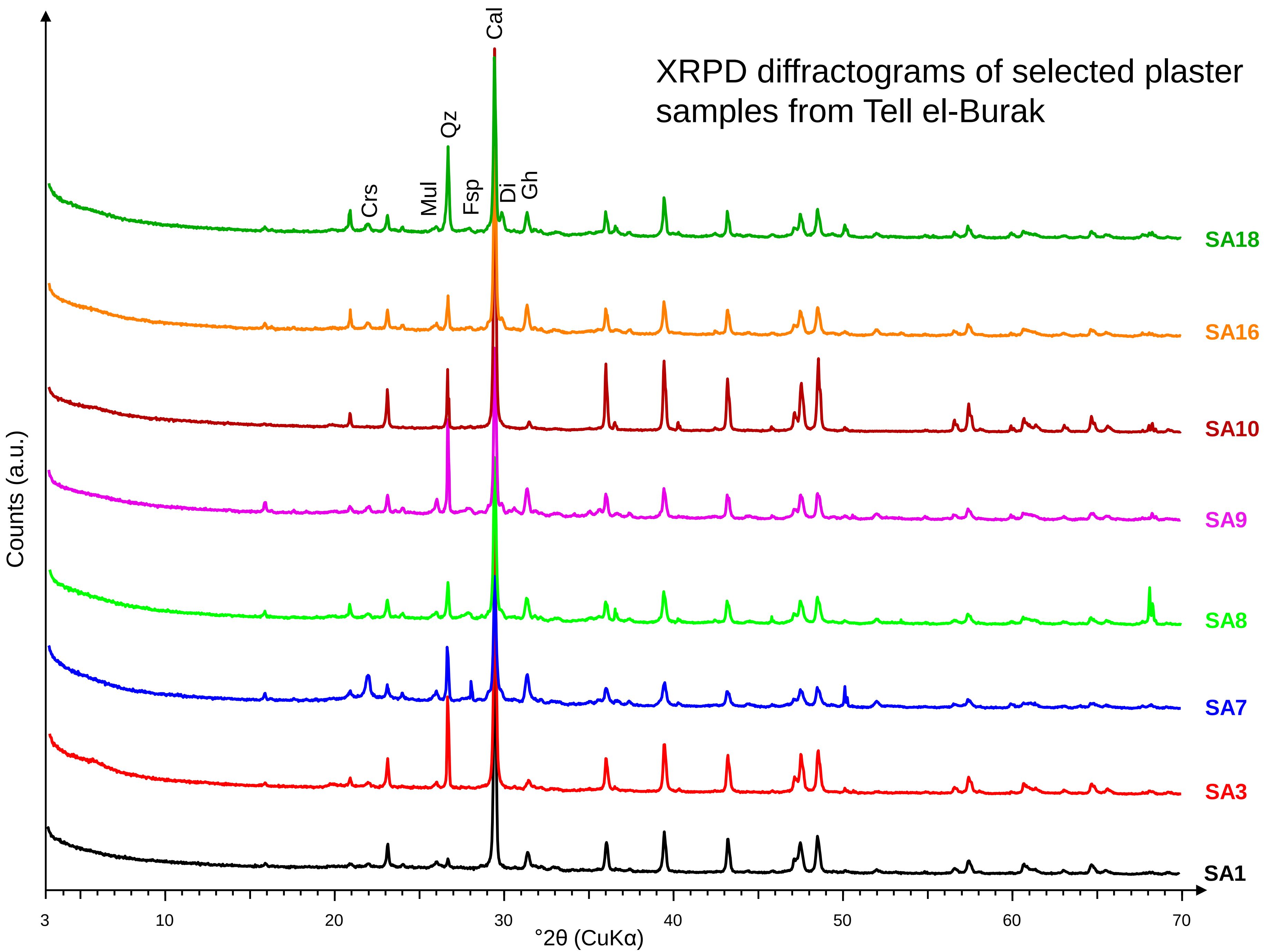
XRPD diffractograms of selected plaster samples from Tell el-Burak
Cal
Qz
Crs
Mul
Fsp
Di
Gh
SA18
SA16
SA10
SA9
SA8
SA7
SA3
SA1
Counts (a.u.)
°2θ (CuKα)
3
10
20
30
40
50
60
70

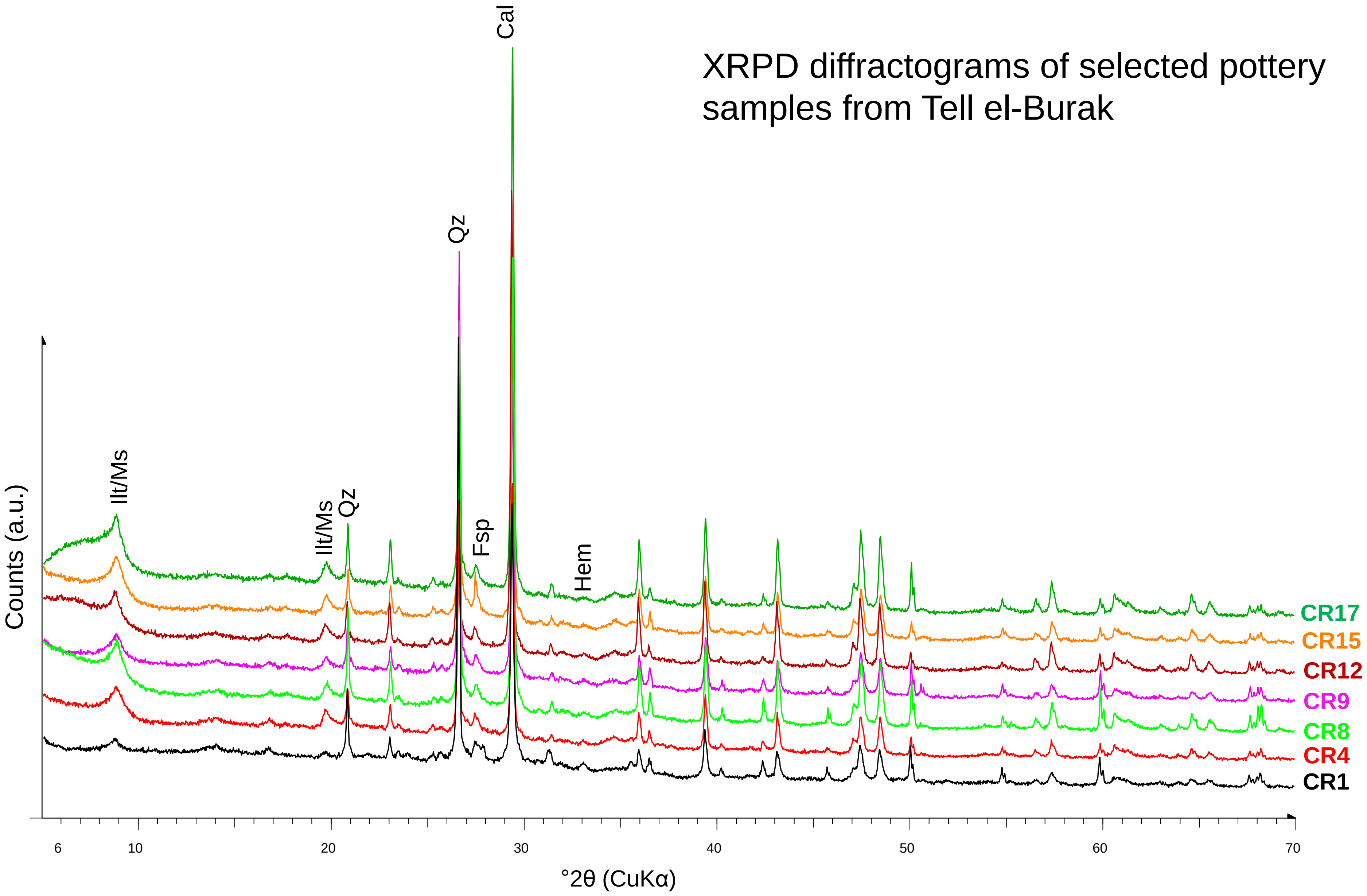
XRPD diffractograms of selected pottery samples from Tell el-Burak
Cal
Qz
Ilt/Ms
Ilt/Ms
Qz
Fsp
Hem
CR17
CR15
CR12
CR9
CR8
CR4
CR1
Counts (a.u.)
6
10
20
30
40
50
60
70
°2θ (CuKα)

X-ray microdiffraction (μ-XRD²) diffractograms of selected plaster samples from Tell el-Burak

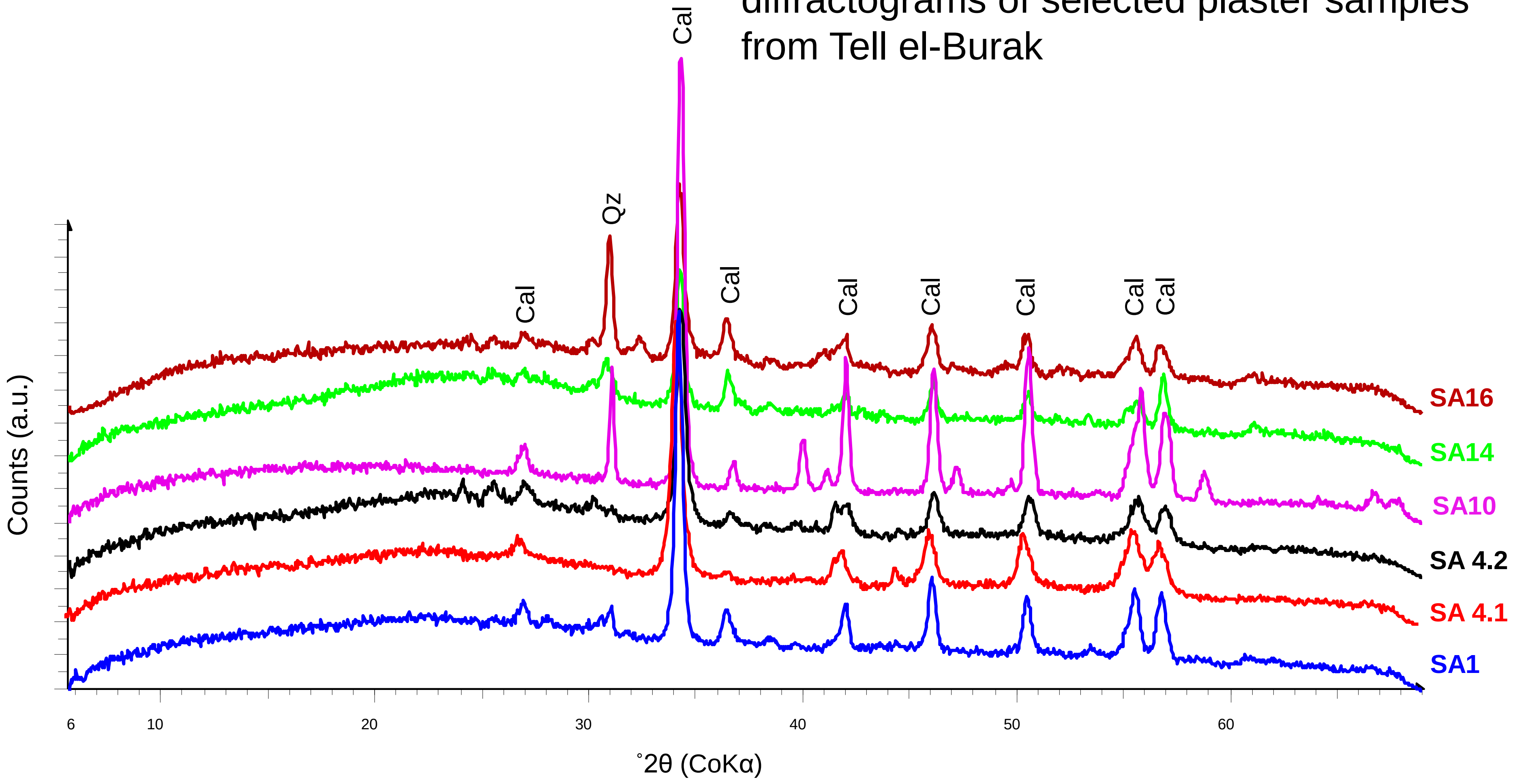

From: Orsingher, A., Amicone, S., Kamlah, J., Sader, H. & Berthold, C. Phoenician lime for Phoenician wine: Iron Age plaster from a wine press at Tell el-Burak, Lebanon. Antiq. 377, 1224-1244; DOI: https://doi.org/10.15184/aqy.2020.4 (2020).
