# Supplementary material for: Innovation through recycling in Iron Age plaster technology at Tell el-Burak, Lebanon
Source: Sci Rep. 2025 Jul 7;15:24284. doi: 10.1038/s41598-025-05844-x (PMC12234981; doi:10.1038/s41598-025-05844-x)

Table 1 SI 3. Semi-quantitative SEM-EDS micro-chemical analyses of selected points from SA1, SA3, SA4, SA6, SA8, SA10. Areas and point analyses of lime lumps, binders, reaction rims, ceramic fragments, limestone fragments (rock for lime) and bioclast fragments in limestone were collected. Hydraulicity index (HI) was calculated for lime lump, binder and reaction rim. A selection of relevant spots analysis (Sp) are reported in Figure 1 SI 3. T1=Type 1 ceramic; T2=Type 2 ceramic

ID sample	NaO	MgO	Al_2O_3	SiO_2	P_2O_5	SO_3	K_2O	CaO	TiO_2	Fe_2O_3	Total	HI
**SA1**												
Lime Lump		1.6	1.0	4.4	1.0	1.5		90.5			100.0	0.06
Lime Lump		1.1	0.4	2.7	1.1	0.7		93.9			100.0	0.03
Lime Lump		1.6		2.7		0.5		95.2			100.0	0.03
Lime Lump		1.6	0.0	4.0		1.0		93.3			100.0	0.04
Reaction rim area T1	1.2	3.0	5.4	33.2	5.2	1.9	0.6	47.2		1.5	99.3	0.80
Ceramic fragment T1	1.5	1.6	9.7	57.1	6.9	3.0	0.5	17.4		1.7	99.5	-
Ceramic fragment T1	0.8	2.3	8.4	55.8	5.4	2.3		20.0		3.9	98.9	-
Ceramic fragment T1	1.7	4.5	10.0	71.2		3.8	0.6	4.8		3.0	99.4	-
Ceramic fragment T1	1.1	1.7	11.1	42.6		2.5	0.0	33.6		7.3	100.0	-
Ceramic fragment T1		2.4	20.0	58.4		1.1	1.8	3.6	1.2	11.5	100.0	-
Ceramic fragment T1		3.4	23.3	56.0		0.9	2.0	2.7	1.1	10.7	100.0	-
Ceramic fragment T1		2.0	18.5	57.2			2.1	5.6		14.7	100.0	-
Ceramic fragment T1		2.9	21.0	54.7		0.8	2.0	4.8	1.5	12.4	100.0	-
Ceramic fragment T1	0.6	3.1	23.4	57.6		1.3	1.7	3.3	0.8	8.1	100.0	-
Ceramic fragment T2		1.0	3.9	85.0		0.7		7.3		2.1	100.0	-
Ceramic fragment T2	0.8	2.4	16.2	53.5		2.7	3.9	13.3	1.3	5.8	100.0	-
Ceramic fragment T2	1.2	2.8	13.8	52.1			3.9	19.8		6.4	100.0	-
Ceramic fragment T2	1.0	1.9	16.7	46.5	0.6	2.2	4.9	19.3	0.9	5.7	99.7	-
Ceramic fragment T1	1.8	2.0	17.0	48.5	0.6	1.0	6.0	16.5	0.7	5.4	99.5	-
Ceramic fragment T1	1.5	2.4	17.7	52.5			5.6	14.5		5.9	100.0	-
Ceramic fragment T1	1.5	2.5	18.5	49.1			5.7	16.3		6.3	100.0	-
Ceramic fragment T1	1.7	2.4	12.4	49.0	1.8	1.6	2.5	22.0		6.5	100.0	-
Ceramic fragment T1	2.0	2.1	8.4	40.7	3.0	1.4	0.8	38.4		2.4	99.2	-
Quartz				99.4				0.6			100.0	-
**SA3**												
Limestone fragment area		1.6	1.3	10.8		1.3		85.0			100.0	-
Limestone fragment area	2.1		6.7	11.5	0.8	1.4	0.6	73.6		3.0	100.0	-
Limestone fragment		1.4	2.2	3.8	7.0	2.5		70.9		1.1	100.0	-
Ceramic fragment T2	5.4	3.0	20.0	56.5			0.3	7.9	1.5	5.3	99.8	-
Ceramic fragment T2	4.9	2.2	18.6	52.0			1.5	15.8	0.8	4.0	99.8	-
Ceramic fragment T2	2.2	2.4	19.0	57.5		1.3	6.8	3.3	0.8	6.3	99.6	-
Ceramic fragment T2	1.4	2.1	11.1	67.2			2.2	9.1		6.7	99.7	-
Ceramic fragment T2	1.8	7.6	16.3	58.4		1.6	2.0	2.0	1.9	8.0	99.5	-
Reaction rim T2		1.1	1.1	9.0	0.7	1.8		85.0		1.4	100.0	0.13
Reaction rim T2		1.9	5	32.1	2.5	5.1		49.5		2.2	98.3	0.77
Ceramic fragment T2	4.6	1.7	11.7	69.1			0.6	1.5	0.9	10.0	100.0	-
Ceramic fragment T2	7.4	0.7	19.8	62.6			1.2	1.7	1.0	5.6	100.0	-
Ceramic fragment T2	5.5		23.4	60.8			1.3	1.5	2.9	4.7	100.0	-
Ceramic fragment T2	3.2	0.9	16.1	57.6		1.4	1.4	13.9		5.5	100.0	-

**SA4**												
Lime lump (Sp 1)	0.8	1.2		0.9	1.0	2.0		94.2			100.0	0.01
Lime lump (Sp 2)	0.5			2.0		0.9	0.4	96.3			100.0	0.02
Lime lump (Sp 3)	0.4		0.4	2.1		0.8	0.5	95.8			100.0	0.03
Lime lump (Sp 4)		1.0		0.6	0.6	2.5		94.2			100.0	0.01
Lime lump (Sp 5)		1.1		0.5		3.1		95.3			100.0	0.01
Binder (Sp 6)		0.9	0.8	4.5	1.5	4.8		87.0			99.5	0.06
Binder		0.8	1.2	8.5	1.2	4.5		83.3			99.4	0.11
Binder		0.9		3.0	1.4	4.4		89.7			99.4	0.03
Binder (Sp 7)		1.6		5.6		5.8		85.8			98.8	0.06
Binder		1.5	1.1	7.2	1.1	3.0		86.1			100.0	0.10
Binder		1.2	0.7	5.0	0.8	3.3		89.1			100.0	0.06
Binder		1.2	1.7	11.9	1.1	5.3		78.1			99.2	0.17
Binder		0.7		2.1	1.0	1.2		95.1			100.0	0.02
Binder	0.7	1.1	1.9	7.6				87.5			100.0	0.11
Binder	1.1	1.5	0.6	4.6	1.1	1.8		89.4			100.0	0.06
Binder		1.5	2.1	6.0		1.2		88.1		1.2	100.0	0.10
Binder	0.9	0.8		3.4	0.6	1.6		92.6			100.0	0.04
Binder	1.1	0.8	0.6	2.8		2.9		91.8			100.0	0.04
Reaction rim (Sp 8) T2		2.7	1.7	13.2	0.6	4.1		77.7			100.0	0.19
Reaction rim (Sp 9) T2	0.0	0.7	0.7	22.7	0.7	5.3		69.0			99.2	0.34
Reaction rim T2		1.7	3.5	22.1	0.9	5.8		65.8			100.0	0.38
Reaction rim T2		2.1	3.1	22.6	0.7	2.4	0.4	67.6		1.1	100.0	0.39
Reaction rim T2		1.5	4.2	13.0		1.5	0.5	77.2		2.1	100.0	0.24
Reaction rim T2	0.6	1.0		25.2	0.7	1.7		70.9			100.0	0.35
Reaction rim area T2	0.8	1.9	3.6	24.6	2.2	6.4	0.6	59.3			99.3	0.46
Reaction rim area T2		1.7	1.4	13.5	1.1	5.6		76.2			99.6	0.19
Ceramic fragment (Sp 10) T2	10.2	0.6	21.7	59.8				1.7	1.0	5.0	100.0	-
Ceramic fragment (Sp 11) T2	7.2	0.8	22.2	55.9		0.4		5.8	1.1	6.5	100.0	-
Ceramic fragment T2	4.3	3.4	14.3	39.9			0.2	30.0	0.6	7.3	100.0	-
Ceramic fragment T2 (Sp 12) T2	10.2	0.4	21.6	59.4		0.4		1.9	1.1	5.0	100.0	-
Ceramic fragment T1	0.5	1.4	4.1	77.6		4.9	0.9	4.3		5.3	99.1	-
Ceramic fragment T1		2.1	6.4	53.3		5.5	0.5	28.6		3.0	99.4	-
Ceramic fragment T1	3.1	1.8	13.7	33.0	0.5	0.6	0.3	40.9	0.9	5.2	100.0	-
Ceramic fragment T1	4.7	1.9	19.1	54.9		1.1	0.4	11.4	0.8	5.7	100.0	-
Quartz				100.0							100.0	-
Area with S				1.5		98.5					100.0	-
Area with S			4.7	21.2	9.0	17.4		7.7		6.2	66.3	-
**SA6**												
Lime lump		0.8		4.8	1.0	1.2	0.3	90.5			100.0	0.05
Lime lump		1.1	0.7	5.1				93.1			100.0	0.06
Binder	0.7	2.0	1.9	17.0	3.5	2.3	0.4	72.0			100.0	0.25
Area with S		1.1	1.4	8.1		87.5		1.9			100.0	-
**SA8**												
Bioclast fragment (Sp 1)						0.7		99.3			100.0	-

Limestone fragment (Sp 2)		1.5	2.8	9.8		1.7		84.2			100.0	-
Ceramic fragment (Sp 3) T2	0.4	3.4	14.3	57.9	1.1	1.1	10.6	6.4		4.8	100.0	-
Ceramic fragment (Sp 4) T2	0.5	1.9	11.9	50.4			10.4	15.7	1.3	7.7	100.0	-
Ceramic fragment (Sp 5) T2	0.6	2.2	16.6	41.3		4.5	1.5	26.1	0.8	6.5	100.0	-
Ceramic fragment T2	2.1	2.1	13.7	48.5		6.7	2.6	13.0	1.3	8.2		-
**SA10**												
Lime lump (Sp 1)	0.7	1.4	1.1	7.9	0.6	3.5	0.6	83.0		0.8	99.6	0.12
Lime lump (Sp 2)	0.7	1.3	1.1	7.7	0.9	3.5	0.4	83.9			99.4	0.10
Binder (Sp 3)	0.5	1.4	3.7	22.4	1.3	11.5		56.5			97.4	0.45
Binder	0.9	3.2	5.0	27.9	2.0	7.2	0.6	51.3		1.0	99.1	0.62
Reaction rim area** (A 1) T1	0.7	1.6	10.6	38.4	0.5	15.3	1.9	26.0	0.6	3.9	99.4	-
Reaction rim area** T1	1.0	1.8	12.3	42.7	1.0	7.3	1.0	28.6		3.8	99.3	-
Ceramic fragment (Sp 4) T1	0.8	1.7	11.0	39.9		16.1	2.0	22.9	0.7	4.3	99.4	-
Ceramic fragment (Sp 5) T1	1.4	1.6	12.5	46.1	0.7	8.1	1.5	23.1	0.7	3.7	99.1	-

*HI index was calculated by Boynton formula[22].

**inner portion of the ceramic fragment.

Figure 1 SI 3. A selection of backscattered electron images of analyzed samples SA4, SA8, SA10

SA4: a) ceramic fragment T2, b) lime lump; SA8: c) limestone fragment with bioclast (left) and ceramic fragment (with reaction rim (right)) T2; SA10: d) lime lump, e) ceramic fragment with reaction rim T1.

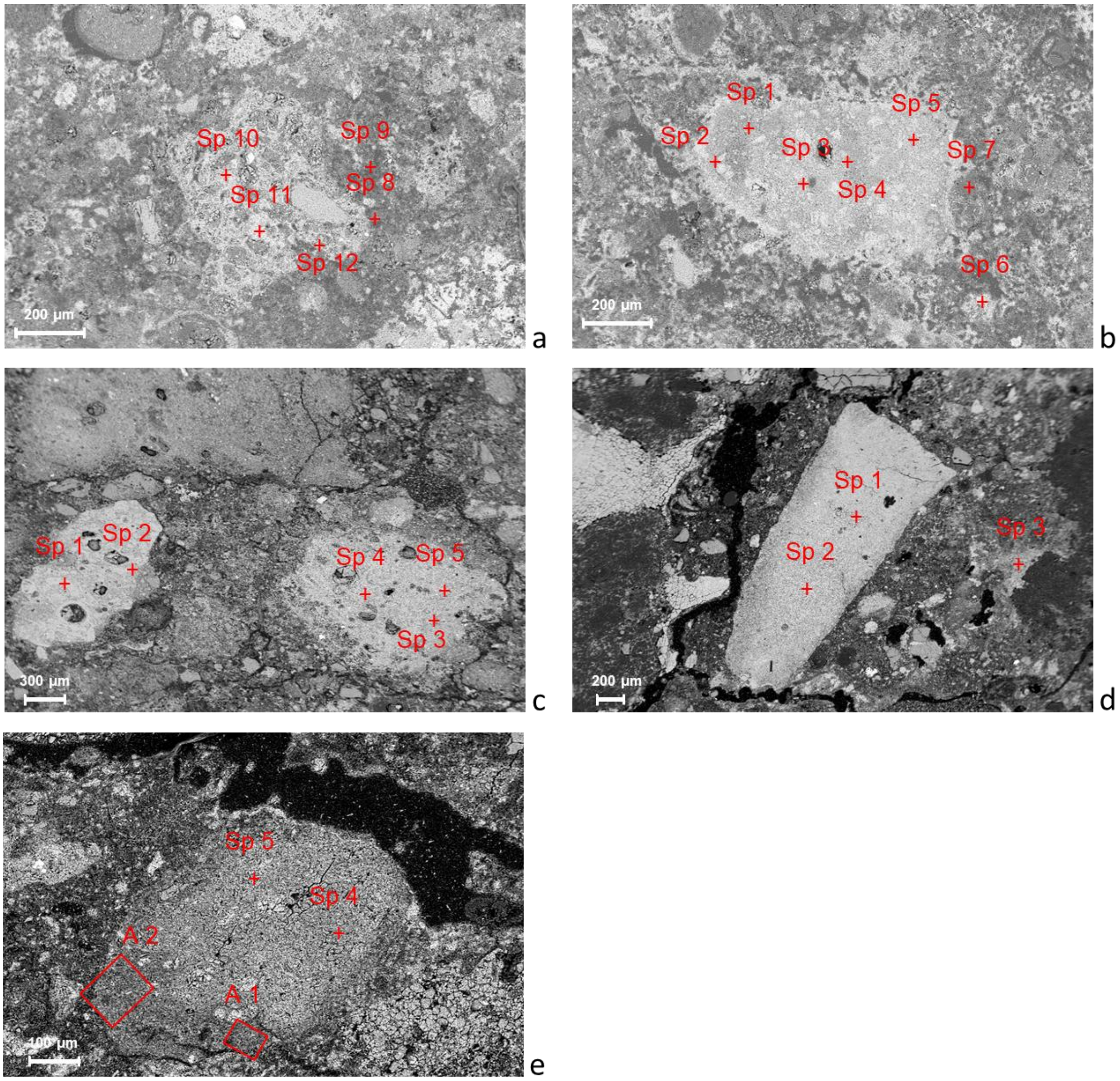

Figure 2 SI 3. Semi quantitative micro-chemical analyses of SiO_2, Al_2O_3, Fe_2O_3, CaO and MgO (% oxides) performed on a line from the binder to the ceramic fragment of sample SA1.(Type 1)

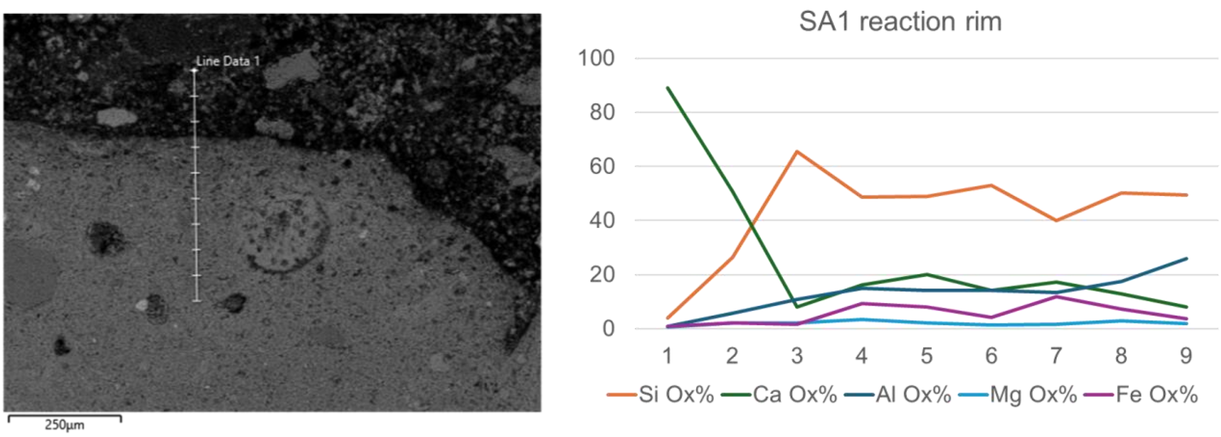

Supplement: Supplementary file 3 — Supplementary Material 3 [file 41598_2025_5844_MOESM3_ESM.pdf]
